# Positive and negative plant−plant interactions influence seedling establishment at both high and low elevations

**DOI:** 10.1007/s00035-023-00302-8

**Published:** 2023-11-24

**Authors:** Chantal M. Hischier, Janneke Hille Ris Lambers, Evelin Iseli, Jake M. Alexander

**Affiliations:** Institute of Integrative Biology, ETH Zürich, 8092 Zürich, Switzerland

**Keywords:** Facilitation, Competition, Stress gradient hypothesis, Vital rates, Range shift

## Abstract

Deciphering how plants interact with each other across environmental gradients is important to understand plant community assembly, as well as potential future plant responses to environmental change. Plant**−**plant interactions are expected to shift from predominantly negative (i.e. competition) to predominantly positive (i.e. facilitation) along gradients of environmental severity. However, most experiments examine the net effects of interactions by growing plants in either the presence or absence of neighbours, thereby neglecting the interplay of both negative and positive effects acting simultaneously within communities. To partially unravel these effects, we tested how the seedling establishment of 10 mountain grassland plants varied in the presence versus absence of plant communities at two sites along an elevation gradient. We created a third experimental treatment (using plastic plant mats to mimic surrounding vegetation) that retained the main hypothesised benefits of plant neighbours (microsite amelioration), while reducing a key negative effect (competition for soil resources). In contrast to our expectations, we found evidence for net positive effects of vegetation at the low elevation site, and net negative effects at the high elevation site. Interestingly, the negative effects of plant neighbours at high elevation were driven by high establishment rates of low elevation grasses in bare soil plots. At both sites, establishment rates were highest in artificial vegetation (after excluding two low elevation grasses at the high elevation site), indicating that positive effects of above-ground vegetation are partially offset by their negative effects. Our results demonstrate that both competition and facilitation act jointly to affect community structure across environmental gradients, while emphasising that competition can be strong also at higher elevations in temperate mountain regions. Consequently, plant**−**plant interactions are likely to influence the establishment of new, and persistence of resident, species in mountain plant communities as environments change.

**Supplementary Information:**

The online version contains supplementary material available at 10.1007/s00035-023-00302-8.

## Introduction

As species shift their ranges upward and poleward in the face of rapidly changing climate, we expect communities to reassemble, with cold-adapted species being extirpated and replaced with warm-adapted species. However, for range and community shifts to occur, plant species must be able to successfully establish into and integrate with resident communities. Seedling establishment is thus a key life-history stage affecting both range shifts and population persistence (Vazquez-Ramirez and Venn 2021), while plant**−**plant interactions play a crucial role in shaping community responses to rapidly changing climate (HilleRisLambers et al. 2013; Alexander et al. 2018). Understanding how plant**−**plant interactions influence warming-induced range shifts is complicated by the fact that interactions span from negative (primarily competitive) to positive (primarily facilitative), meaning that establishment and range shifts could either be slowed or accelerated by the presence of neighbours (Stephan et al. 2021). It is therefore important to understand how the spectrum of facilitative and competitive plant**−**plant interactions vary with the environment, both spatially and temporally (Anthelme et al. 2014; Michalet et al. 2014).

A potentially useful framework for predicting how neighbours influence recruitment is the stress gradient hypothesis (SGH; Callaway et al. 2002; Kikvidze et al. 2011; Soliveres and Maestre 2014), which posits that interactions in abiotically harsh environments are primarily positive, while those in abiotically benign environments are primarily competitive. Specifically, plants at lower elevations are thought to compete intensely for light and nutrients, while at higher elevations they derive a benefit from the amelioration of microclimate by nurse plants or the vegetation as a whole (e.g. buffering temperature extremes; Körner 2003; Anthelme et al. 2014; Liancourt et al. 2020) and from improved below ground conditions, such as increasing nutrient or water availability (Körner 2003; Anthelme et al. 2012, 2014; Lembrechts et al. 2015; Chen et al. 2020). Accordingly, range shifts at high latitude and elevation may be promoted by neighbours (Anthelme et al. 2014; Chen et al. 2020), while those at lower latitude and elevation would be slowed by neighbour interactions (Olsen et al. 2016). However, even in temperate mountain systems, competition can be strong in closed vegetation at high elevation while plants also experience environmental stress at low elevation (e.g. Lyu and Alexander 2022). Furthermore, no matter what environmental conditions they occur under, the outcome of plant**−**plant interactions arises from the totality of negative and positive effects of neighbours acting in concert (Callaway and Walker 1997; Gross et al. 2010; Hart and Marshall 2013; Michalet et al. 2014; Liancourt et al. 2020; Lyu and Alexander 2023). Thus, a deeper appreciation of how plant**−**plant interactions may influence range shifts requires unravelling this joint action of positive and negative interactions (Stephan et al. 2021).

Studies of facilitation increasingly demonstrate that although positive effects may predominate, beneficiary species also suffer from negative impacts (i.e., competition) from their benefactors. For example, increasing cover of beneficiary species can lead to reduced flowering of cushion plants (Schöb et al. 2014; García et al. 2016; Michalet et al. 2016), although this may be offset by positive effects on fruiting (García et al. 2016). Similar costs of facilitation have also been demonstrated to occur in animals (Hart and Marshall 2013; Dangles et al. 2018) and are therefore likely to be general. We should also expect a mixture of both negative and positive effects to be imposed by benefactor species on beneficiary species. For example, a model developed by Lembrechts et al. (2015) illustrates how the net effects of vegetation on seedling recruitment in Arctic**−**alpine ecosystems arise from positive effects on microclimate and resource supply, and negative effects of competition both above and below ground. In this model, plants are therefore expected to establish in gaps that are small enough to provide microclimate amelioration but large enough to diminish effects of competition. Consistent with this model, Anthelme et al. (2017), found that variation in the canopy properties of cushion plants at both intra- and interspecific levels could explain variation in the strength of facilitation. Other empirical studies show that seedling recruitment relies on gap formation in closed tundra vegetation (Klanderud 2010; Graae et al. 2011; Milbau et al. 2013), demonstrating the importance of competitive effects. Thus, to understand how recruitment will be affected by neighbours, both today and as environments change, we must find ways to (at least partially) tease apart the combined action of negative and positive effects.

**Fig. 1:**
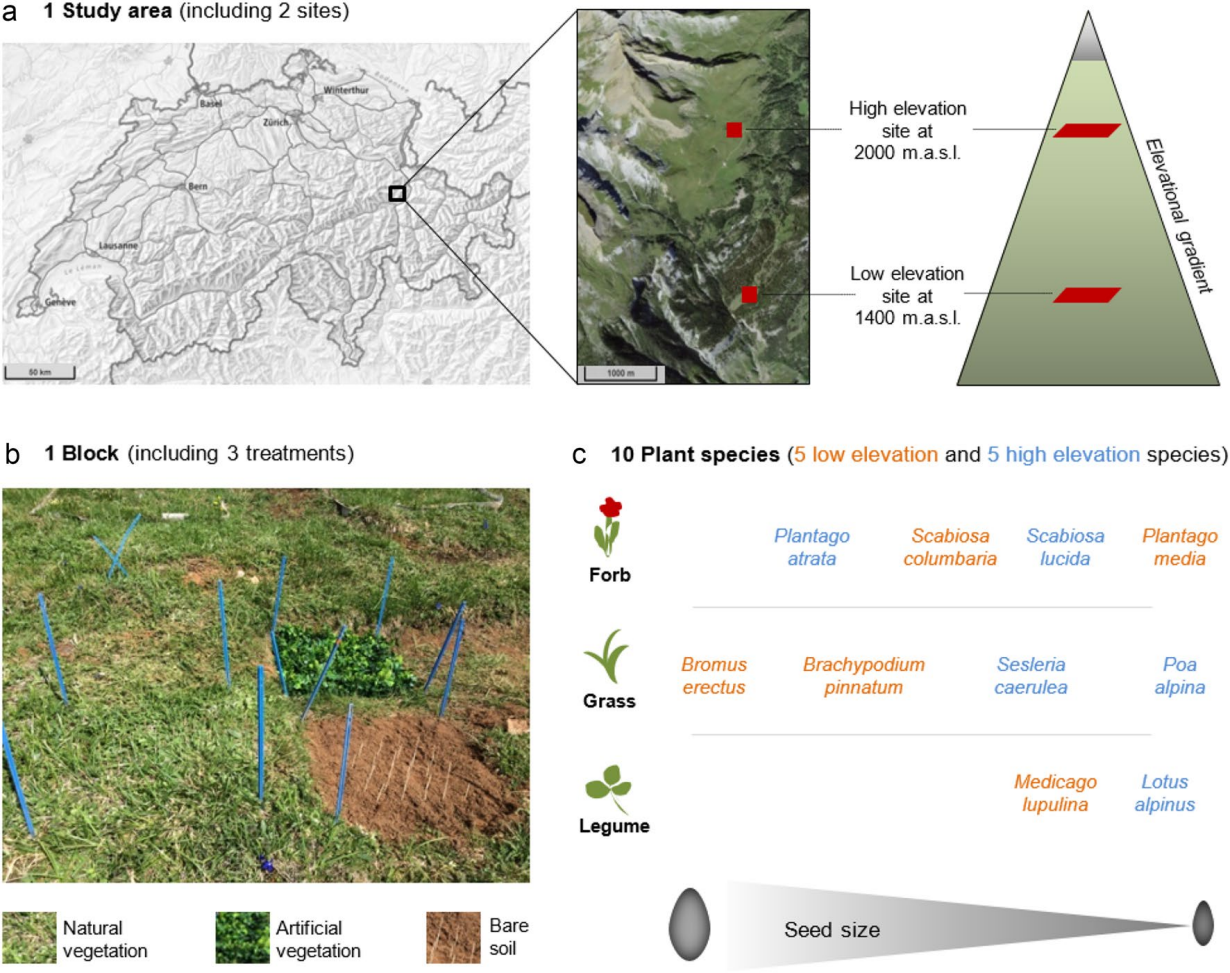
Experimental design and selected focal plant species. The study area on the Calanda, a mountain in the Eastern Swiss Alps, included two sites at 1400 and 2000 m.a.s.l. (**a**; maps retrieved from map.geo.admin.ch). Both sites contained ten blocks, each consisting of one plot per treatment (natural vegetation, artificial vegetation and bare soil; **b**). Seeds from one of ten selected plant species originating from two different elevations (low elevation, high elevation), belonging to three different functional groups (forb, grass, legume) and showing a pronounced seed size gradient (**c**) were glued to wooden toothpicks (*n* = 3 per species and treatment) and planted in each plot (**b**)

Experiments typically examine the net effects of neighbours on a focal species by growing plants in either the presence or absence of neighbours (e.g. Callaway et al. 2002). With such a design, it is not possible to distinguish whether positive and negative effects of neighbours are strong or weak, nor to determine their relative contribution to plant performance. For instance, weak net positive effects of neighbours at high elevation might arise because facilitation is weak and competition is negligible, or because competition is strong but facilitation is stronger. Here, we use a seedling establishment experiment at two sites along an elevation gradient in the Swiss Alps (Fig. 1) in an attempt to disentangle facilitative and competitive community effects on the seedling establishment of lowland and highland plants. To quantify net effects of above- and below-ground competition and facilitation on establishment, seeds were planted into either bare soil plots with vegetation and roots removed, or into existing natural vegetation, respectively. To partially decouple these net positive and negative effects of vegetation, we created a third experimental treatment that retained the main hypothesised benefit of neighbours (microsite amelioration), while reducing a key negative effect (competition for soil resources). To do so, we planted seeds into plots from which natural vegetation and roots had been removed (as for the bare soil treatment) and then replaced with a section of artificial vegetation (Fig. 1). Artificial vegetation sections were commercial plastic vegetation mats intended to approximate shading/microclimate conditions of natural vegetation, consisting of a plastic grid with attached plastic stems and leaves of about 7 cm height and normally used to cover surfaces in gardens. By comparing recruitment on bare soil, in natural and in artificial vegetation, we can learn about the relative intensity of competition and facilitation operating within the plant communities. We acknowledge, however, that a complete separation of these effects is not possible with our (or indeed any) artificial vegetation treatment, as we explore further in the Discussion.

We begin by asking, (1) do the effects of competition and facilitation on seedling establishment vary by elevation? Specifically, we hypothesised that competitive effects would be more negative at low elevations, where vegetation is taller, and facilitative effects more positive at high elevation, where temperatures are lower, such that negative plant** − **plant interactions predominate at low elevations while positive effects predominate at high elevations. This would be reflected in highest establishment on bare soil at low elevation due to release from competition for light and soil resources, followed by artificial vegetation (assuming some facilitative effects are conferred by artificial vegetation). At high elevation, by contrast, we would expect stronger net effects of facilitation, reflected in highest establishment in artificial vegetation due to the retention of microclimate amelioration, and lowest establishment on bare soil due to high abiotic stress. Intermediate establishment success in natural vegetation would reveal the combined action of negative and positive effects of neighbours at high elevation.

We next ask, (2) can variation in seedling establishment across species be explained by species’ elevation of origin, seed size or functional group? It is known that the dependence of seedling establishment on facilitation, or the sensitivity of seedling establishment to competition, may be influenced by functional traits like seed size or plant functional group. For example, larger seed size may allow seedlings to better tolerate competition from closed vegetation or to successfully compete for access to establishment sites (Goldberg and Landa 1991; Turnbull et al. 1999; Coomes and Grubb 2003), and therefore might also mediate the dependence of establishment on facilitation. We hypothesise that small-seeded species depend to a greater extent on positive effects of vegetation for microclimate amelioration/protection from environmental hazards at high elevation but are also more sensitive to competition (and so show proportionally greater establishment in artificial vegetation). In contrast, species possessing large seeds may better tolerate environmental hazards by virtue of larger maternal provisioning (Coomes and Grubb 2003; Ben-Hur et al. 2012; Maron et al. 2019), and consequently depend less on facilitation for establishment at high elevation. Greater maternal provisioning may also confer large seeded species with a greater tolerance of competition, especially at low elevation, although evidence that larger seeds (and hence seedlings) have greater competitive ability is mixed (Coomes and Grubb 2003; Ben-Hur and Kadmon 2015). In addition to seed size effects, plant responses to neighbours could vary between functional groups. For example, Schöb et al. (2018) suggested that legumes might profit more from facilitation than herbs and grasses.

## Material and methods

### Study system and plant species

To disentangle positive and negative effects of plant**−**plant interactions on seedling establishment, we conducted a field experiment in the Eastern Alps of Switzerland at two elevations (1400 m.a.s.l., 46.869 °N, 9.490 °E and 2000 m.a.s.l., 46.888 °N, 9.489 °E) on the Calanda mountain over a five-month period (June** − **October 2021). Both sites are perennial grassland sites dominated by compact vegetation (approx. 100% vegetation cover) and managed as low intensity summer pasture for cattle, located on calcareous bedrock (Alexander et al. 2015) and show both south-western exposure and comparable slope. The low elevation (montane) site experiences warmer, drier and longer summers, while the high elevation (subalpine, i.e. below climatic treeline; Körner 2021) site is located on a wind-exposed plateau above the timberline and experiences a cooler climate (see Fig. S1 for conditions during the experiment). The mean annual temperature between 2000 and 2016 was 4.85 ± 0.06 °C (mean ± SD) at the low elevation site and 2.07 ± 0.56 °C at the high elevation site, while the mean annual precipitation sum was 981.41 ± 117.94 mm and 1123.94 ± 135.35 mm (based on CHELSAcruts monthly minimum and maximum temperatures and monthly precipitation sums; Karger and Zimmermann 2018). The low elevation site is characterised by quite dense and tall herbaceous vegetation (typical species *Brachypodium pinnatum, Salvia pratensis, Carlina acaulis, Plantago media*), while the high elevation site hosts a low-growing and herbaceous vegetation cover (typical species *Sesleria caerulea, Alchemilla conjuncta, Anthillis vulneraria, Thymus* spp.). Both experimental sites were fenced to exclude livestock.

Ten focal plant species were selected, including five low elevation (*Plantago media*, *Scabiosa columbaria*, *Bromus erectus*, *Brachypodium pinnatum*, *Medicago lupulina*) and five high elevation species (*Plantago atrata*, *Scabiosa lucida*, *Poa alpina*, *Sesleria caerulea*, *Lotus alpinus*) that naturally occur at the 1400 m.a.s.l. and 2000 m.a.s.l. sites, respectively (Table S1). These species were selected to include a variety of functional groups (graminoids, leguminous forbs, nonleguminous forbs), whilst controlling for phylogenetic effects by equally representing families within the low- and high-elevation groups, and to cover a gradient of seed size in each group (Fig. 1). Seeds of low and high elevation species were obtained from commercial suppliers (from UFA-Samen and Otto Hauenstein Samen, respectively), selecting seeds from the local Canton des Grisons ecoregion wherever possible.

### Experimental design and environmental conditions

At each site we established ten blocks within an area (ca. 100–200 m^2^) of approximately homogenous terrain to control for variation in vegetation composition and microtopography. Blocks contained three 42 × 42 cm plots that were assigned at random to three experimental treatments (natural vegetation, artificial vegetation and bare soil; Fig. 1). Plots within a block were 15 cm apart while blocks were at least 40 cm apart (except for one case with only 20 cm between two blocks). To create the natural vegetation treatment, plots were left with natural vegetation intact, and therefore retained all negative and positive effects of the vegetation on focal plants. To create the bare soil treatment (neither positive nor negative effects of vegetation), the vegetation and the top ca. 10 cm of soil with roots were removed from plots and replaced with soil from the same elevation. This was repeated to create the artificial vegetation treatment, except that in addition to refilling the plots with soil, we fixed a 42 × 42 cm artificial vegetation mat on top of the plot. The artificial vegetation is normally used as a covering for surfaces in gardens (purchased from JUMBO, product number: Art.1369297), and comprises plastic “leaves” attached to a plastic grid (ca. 2.5 cm), to a height of approximately 7 cm, similar to the vegetation height at our high elevation site. The grid allowed spaces for seeds to be planted within the “vegetation”, while the artificial plants simulated the aboveground vegetation itself (Figs. S2, S3). The artificial plants provided a relatively dense neighbourhood, which we quantified in terms of light interception at soil level (see next paragraph). We imposed the artificial vegetation treatment to mimic aboveground effects of the plant community on seedlings (especially facilitation through microclimate amelioration, but also e.g. competition for light), while omitting most below ground effects (especially competition for water and nutrients, but also e.g. microbial community effects).

To determine treatment-related differences in environmental parameters, we measured soil temperature and moisture in the plots as well as light intensity at the soil surface. Six temperature loggers (HOBO UA-002-64 Pendant Temperature/Light 64 K Data Logger; www.onsetcomp.com) were buried with the upper edge 1 cm below the soil surface in six plots (two per treatment) at each site in mid-June 2021 to measure soil temperature every 30 min until late October. Soil moisture (volumetric water content [%]) was measured in all plots at both sites on one day in the beginning of September 2021, using a time-domain reflectometer (TDR) across a 5.3 cm soil profile. Soil moisture was measured as the average soil moisture of ten evenly distributed measurement points in every plot, ensuring that plot heterogeneity was accounted for. Light interception by natural and artificial vegetation was measured as the difference in light incidence (µmol m^−2^ s^−1^) at 50 cm above and immediately at the soil surface for 10 measurement points per plot. Light measurements were carried out on a sunny day towards the end of the season (1st September 2021) with stable weather and light conditions, and the light sensor was always aligned parallel to the ground at the same angle while measuring.

Environmental variables were analysed using linear mixed effects models (LMM) using the “lme4” package (Bates et al. 2015) to test for differences between treatments, sites and their interaction. The LMM for soil temperature used all observations from the 12 HOBO loggers (*n* = 5640–5650 per logger) and included logger ID as a random effect. The LMM for soil volumetric water content contained plot as a random effect, while the LMM for percent light interception (i.e., [light at ground level/light at 50 cm] * 100) contained both plot and block random effects and excluded the bare soil treatment. We square-root transformed temperature and light interception data to improve homogeneity of variance after visually checking diagnostic plots, and in all models determined statistical significance based on Type II Wald χ^2^ tests from the “car” package (Fox and Weisberg 2019).

Across all treatments, mean daily soil temperature (measured from 18 June to 25 October) was 3.65 °C higher at the low as compared to the high elevation site; temperatures were highest in bare soil plots and similar in natural and artificial vegetation (Fig. S1; Tables S2, S3). Soil moisture was higher at the high elevation site and in natural vegetation at both sites, with similarly low moisture availability in artificial vegetation and bare soil, especially at the low elevation site (Fig. S1; Tables S2, S3). There was also significantly greater light interception by natural than artificial vegetation, although both treatments reduced light to a similarly large extent (to 4.1% vs 2.1% of ambient, respectively) and this effect did not differ significantly between the low and high elevation sites (Tables S2, S3). We thus conclude that our artificial vegetation treatment was effective at simulating microclimate conditions and aboveground competition for light in natural vegetation, while removing other biotic effects belowground.

### Assessment of seedling establishment

Seeds were sown by first gluing them to toothpicks using water-soluble PVA glue to avoid them being washed away or removed by seed predators prior to germination (Fig. S2). Toothpicks were first dipped in diluted PVA glue and afterwards into a vial containing seeds of a given plant species, leaving an approximately equal number of seeds stuck to each toothpick. We made counts of seeds stuck to 20 toothpicks per species as an estimate of the initial seed number, as this number varied by species and was important to assessing germination rates (proportion of seeds observed as seedlings). Three toothpicks per species were inserted in a predefined position (“station”) at 5 cm apart within a grid overlaid on each plot, yielding a total sample size of *N* = 180 per species (3 replicates × 10 blocks × 3 treatments × 2 sites) or *N* = 1800 replicates across all species. In the artificial vegetation treatment, toothpicks were inserted into the plastic grid interstices between the plastic plants. The position of the plant species within a plot was randomised (with the same order in every plot), but with the condition that each species occurred no more than two times in the outer rows of the grid to avoid uneven edge effects across species. Each plot contained a 10 cm-buffer around the outer row of stations to minimise edge effects.

To record seedling establishment, we counted the number of seedlings at each station in all plots every 2–3 weeks (in total eight times) during the vegetation period from June to October 2021. A seedling was counted as soon as the cotyledons were visible. Therefore, we consider “seedling establishment” to include both successful germination and initial seedling growth. Seedlings were not thinned out during the vegetation period. To determine the average seed mass of each plant species, we weighed ten batches of 50 seeds of each plant species and then calculated the average mass of a single seed per species.

### Statistical analyses

To address our first question, we fitted a base model to data from all species combined to test whether seedling establishment depended on elevation (experimental site), treatment (natural vegetation, artificial vegetation, bare soil), and their interaction. We fitted a generalised linear mixed effects model (GLMM) using the BOBYQA optimiser in the lme4 package (Bates et al. 2015). Establishment rate was modelled as a binomial response variable (counts of “successes” and “failures”), where “successes” were the maximum number of seedlings observed across all eight censuses at a given station, and “failures” were calculated as this number minus the mean initial number of seeds per toothpick for that species (see above). “Species” and “Plot” were included as random effects to account for the nestedness of observations; in exploratory models the “Block” term resulted in singular fits (zero variance) and was not included. We also included an observation level random effect to account for overdispersion. To answer our second question (whether seed size, species origin or functional group could explain responses), we modified this base model to investigate whether variation in establishment could be additionally explained by species origin (low or high elevation), seed size (continuous) or functional group (grass, legume, nonleguminous forb). Specifically, because traits were overlapping and the number of species tested low, we added one of these variables as a third fixed effect (i.e., in separate models) to the base model, including all interactions among fixed effects and keeping the response variable and random effects unchanged. The explanatory power of the base model and the three models containing either species origin, seed size or functional group were compared based on the Akaike Information Criterion with a correction for small sample size (AICc). The statistical significance of model terms was determined using Type II Wald *χ*^2^ tests from the “car” package (Fox and Weisberg 2019). All analyses were conducted with R version 1.4.1106 (R Core Team 2022).

## Results

### Do the effects of plant−plant interactions on seedling establishment vary with elevation?

**Fig. 2:**
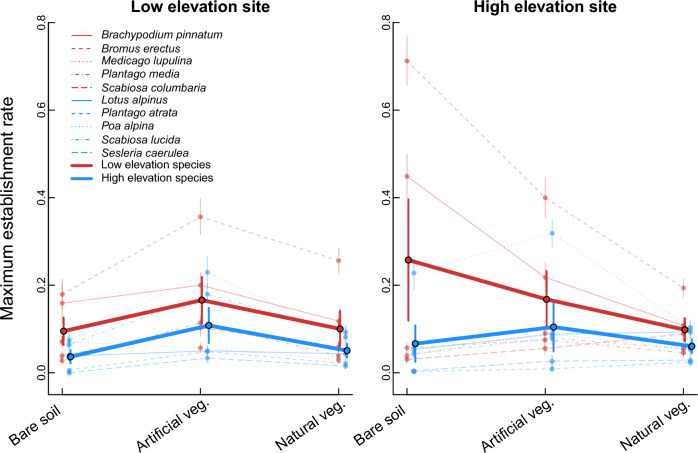
Seedling establishment rate (± standard error) for low (red lines) and high (blue lines) elevation focal species under three experimental treatments (bare soil, artificial and natural vegetation) at two experimental sites at low (1400 m a.sl., left panel) and high (2000 m a.sl., right panel) elevation. The thick lines connect mean establishment rates across all species from a given elevation origin in each treatment

Overall, establishment rates significantly differed between sites (Table 1) and were 35.6% higher at the high (0.126 ± 0.007, mean ± SE) than at the low elevation site (0.093 ± 0.005) across all three treatments. Seedling establishment differed between treatments, but this effect was different at the two sites (significant treatment × site interaction, Table 1). Contrary to our hypothesis, the highest establishment rate across all focal species occurred at the high elevation site in bare soil, followed by artificial and then natural vegetation (Fig. 2). In contrast, at the low elevation site the highest establishment was observed in artificial vegetation followed by natural vegetation and the lowest on bare soil. However, there was considerable variation in establishment among species, which we describe in the next section.

**Table 1:** Results of four models of seedling establishment rate, building on a base model including fixed effects of experimental site (low vs. high elevation), vegetation treatment (natural vegetation, artificial vegetation and bare soil) and their interaction. More complex models additionally include fixed effects of either the elevation of origin of the focal species (low vs. high elevation), their mean seed mass or their functional group (grass, legume, forb), and all interactions. All models were fitted as generalised linear mixed effects models (GLMMs) including species identity and experimental plot as random effects, plus an observation level random effect to control for overdispersion

Model	AICc	Fixed effect	*χ*^2^	*df*	*P*
Base	5724.93	Treatment (T)	46.31	2	< 0.001
		Site (S)	15.77	1	< 0.001
		T × S	26.90	2	< 0.001
Elevation origin	5699.07	Treatment (T)	45.56	2	< 0.001
		Site (S)	15.62	1	< 0.001
		Elevation origin (O)	2.38	1	0.123
		T × S	26.44	2	< 0.001
		T × O	28.87	2	< 0.001
		S × O	1.03	1	0.311
		T × S × O	7.80	2	0.020
Mean seed mass	5657.16	Treatment (T)	45.26	2	< 0.001
		Site (S)	15.66	1	< 0.001
		Mean seed mass (M)	2.04	1	0.153
		T × S	26.07	2	< 0.001
		T × M	34.27	2	< 0.001
		S × M	5.52	1	0.019
		T × S × M	40.36	2	< 0.001
Functional group	5598.50	Treatment (T)	41.74	2	< 0.001
		Site (S)	14.30	1	< 0.001
		Functional group (F)	2.96	2	0.228
		T × S	22.03	2	< 0.001
		T × F	46.64	4	< 0.001
		S × F	46.40	2	< 0.001
		T × S × F	64.09	4	< 0.001

### Are species’ responses to interactions with neighbours explained by elevation of origin, seed size and functional group?

Variation in establishment among species was partly related to species’ origin (significant treatment × site × species origin interaction, Table 1). At both sites, low elevation species on average displayed higher establishment rates (0.120 ± 0.007 at the low elevation site; 0.175 ± 0.011 at the high elevation site) than high elevation species (0.065 ± 0.005 at the low elevation site; 0.077 ± 0.006 at the high elevation site) (Fig. 2). Both low and high elevation species responded similarly to treatments at the low elevation site, with elevated establishment in the artificial vegetation. However, at the high elevation site, low elevation species displayed the highest establishment rate on bare soil, followed by artificial and then natural vegetation. Establishment rate of high elevation species at the high elevation site was highest in artificial vegetation, followed by bare soil and natural vegetation.

**Fig. 3:**
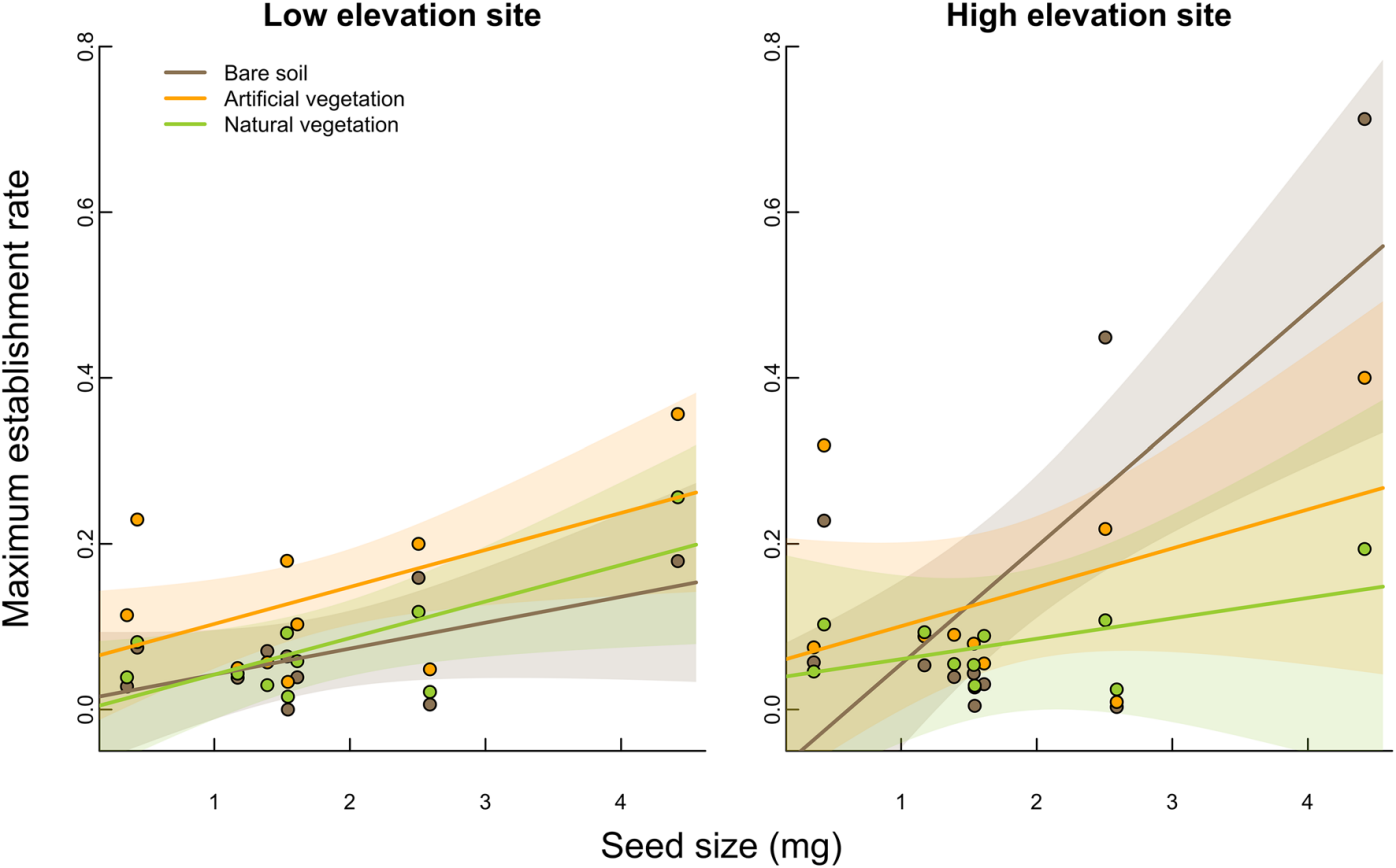
Seedling establishment rate as a function of the mean seed size of focal species under three experimental treatments (bare soil, artificial and natural vegetation) at two experimental sites at low (1400 m a.sl., left panel) and high (2000 m a.sl., right panel) elevation. Trend lines and shaded areas are model fits and their 95% confidence intervals from linear models fitted separately to data from each site

In addition to variation in establishment rate between low and high elevation species, variation in establishment could also be explained by differences among species in their seed size and functional group. Establishment increased with seed size in all treatments at both elevations (Fig. 3). This increase occurred at a similar rate in the natural and artificial vegetation treatments at both the low and high elevation sites and for the bare soil treatment at the low elevation site; however, the relationship between establishment and seed size was much steeper on bare soil at high elevation site (significant treatment × site × seed size interaction, Table 1; Fig. 3).

**Fig. 4:**
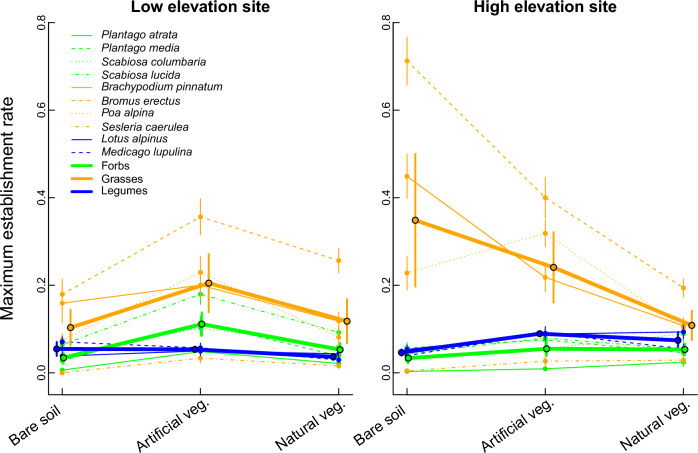
Seedling establishment rate (± standard error) for focal species belonging to the functional groups of forbs (green lines), grasses (orange lines) and legumes (blue lines) at two experimental sites at low (1400 m a.sl., left panel) and high (2000 m a.sl., right panel) elevation. The thick lines connect mean establishment rates across all species from a given functional group in each treatment

Establishment rates also differed significantly between different functional groups, but this also depended on the treatment and site (significant treatment × site × functional group interaction, Table 1; Fig. 4). Grasses had the highest establishment rate across all treatments at both the low (0.142 ± 0.010) and high elevation site (0.232 ± 0.014), followed by forbs (0.066 ± 0.005) and legumes (0.048 ± 0.004) at the low elevation site and legumes (0.070 ± 0.005) and forbs (0.047 ± 0.004) at the high elevation site. However, while forbs and grasses at the low elevation site displayed the highest establishment rate in artificial vegetation and the lowest on bare soil, legumes displayed highest establishment rates on bare soil and the lowest in natural vegetation (Fig. 4). In contrast, at the high elevation site, we observed the highest establishment rate for grasses on bare soil and lowest in natural vegetation. Forbs and legumes established similarly well in artificial and natural vegetation (though slightly better in artificial vegetation) and at the lowest rate on bare soil. The trends for grasses at the high elevation site appeared to be strongly driven by the two well-establishing low elevation species *Brachypodium pinnatum* and *Bromus erectus*. If these species were excluded from the analysis, we found that establishment rate of the different functional groups still differed between treatments and sites (three-way interaction, *χ*^2^ = 24.65, *df* = 4, *P* < 0.001). However, while the patterns for the remaining grasses were unchanged at the low elevation site, at the high elevation site they displayed highest establishment in artificial vegetation followed by on bare soil. Overall, accounting for functional group provided a better fit to the data (i.e. had a lower AIC) than seed size or elevation origin of the focal species (Table 1).

## Discussion

### Competition and facilitation operate jointly across elevation

While current theory emphasises how the net effects of species interactions can vary across broad environmental gradients, ecologists increasingly appreciate that an interplay of facilitative and antagonist effects give rise to overall interaction outcomes. We attempted to partially disentangle some of the positive and negative effects contributing to seedling establishment within vegetation at two sites across an elevation gradient. In contrast to our hypothesis, we found higher competitive effects at high elevation and additionally evidence that facilitative effects could be strong, if species-specific, at both high and low elevations. However, higher performance at high elevations suggests that these patterns may have arisen because stress did not vary in the expected manner with elevation. Such inverted gradients were shown, for example, by Cavieres et al. (2006) in the Andes of central Chile, and it is also known from Mediterranean ecosystems that facilitation can increase towards lower elevations (Michalet et al. 2014). Drought conditions and high soil temperatures at lower elevations might impede the establishment of seedlings that are not afforded protection by surrounding vegetation cover, while also explaining the overall reduction in establishment as compared to the high elevation site. We saw consistent facilitative effects of the artificial vegetation treatment at the low elevation site, which could be explained by a buffering of soil temperature (which was comparable to the natural vegetation; Fig. S1); there was however limited facilitation via soil moisture retention, since values were similar to the water content of bare soil at the low elevation site, although we lack data on humidity to inform whether evapotranspirative stress may have been greater in bare soil plots. Interestingly, establishment rates in natural vegetation and on bare soil were comparable, suggesting that these putative positive effects of vegetation were balanced by negative effects. Presumably competition for light was not a strong constraint since light reduction was comparable in both natural and artificial vegetation. Rather, competition for resources or space belowground likely explains the overall neutral effects of vegetation cover on establishment rates.

A similar interplay of negative and positive effects of vegetation on establishment were observed at the high elevation site. High elevation species displayed greatest establishment in artificial vegetation, and comparable establishment rates on bare soil and in natural vegetation, as also seen at the low elevation site. Thus, positive effects of vegetation on microclimate and soil conditions were offset by its negative effects belowground. In contrast to its effects at the low elevation site, the artificial vegetation at the high elevation site created soil moisture conditions that were intermediate yet more similar to bare soil than natural vegetation, and cooler temperatures than either treatment. The treatment therefore likely underestimated the benefits of vegetation on establishment. At the same time, competitive effects of high elevation vegetation for soil resources must have been correspondingly large to lead to the net effects we observed (Klanderud 2010; Graae et al. 2011; Milbau et al. 2013). In contrast, net effects of high elevation vegetation on low elevation species were overwhelmingly negative, with establishment rates highest in bare soil (no competition) and lowest in natural vegetation (belowground and aboveground competition). While this underscores that competition can also be a strong force in closed vegetation at high elevation (Lyu and Alexander 2022), there was substantial variation among species, which we discuss next.

### Seed size and functional groups explain variation in establishment success

Seed size is an important trait affecting both dispersal strategy and seedling establishment, via maternal investment in seed resources supporting early seedling performance (Moles and Westoby 2004; Ben-Hur et al. 2012), likely explaining why some of the observed variation in species’ establishment success was related to seed size. We generally observed an increase in establishment rate with increasing seed size and this trend was especially pronounced on bare soil at high elevation (Fig. 3), consistent with large seed size promoting establishment and survival in the face of environmental hazards (Coomes and Grubb 2003; Moles and Westoby 2004). Our results however indicate that large-seeded species, at least at high elevation, suffered disproportionately from interactions with intact vegetation. This may indicate a strong effect of competition on large-seeded species by vegetation at the high site, as described above, and/or a strong effect of facilitation on small-seeded species. However, competition for light likely plays a role given the suppression of establishment by both natural and artificial vegetation relative to establishment on bare soil. These patterns also suggest that competition**−**colonization trade-offs are not a strong feature in this system (Turnbull et al. 2004), which would predict proportionally greater establishment of small-seeded species on the bare soil treatment under the assumption that small-seeded species are poor competitors.

The high establishment rates of lowland, large-seeded species on bare soil at high elevation was driven by the two grasses *Brachypodium pinnatum* and *Bromus erectus*. Unsurprisingly therefore, differences between functional groups provided the best explanation of variation in establishment rates (Table 1). Although small sample size (in terms of species number) can influence model effects, the significant three-way interaction between elevation, treatment and functional group remained when fitting a model with these two species excluded, indicating that functional group differences in establishment were not merely driven by these two species. Consistent with Schöb et al. (2018), we found that legumes profited most from facilitation at high elevation, showing increasing establishment in both natural and artificial vegetation with increasing elevation. This role of legumes as beneficiaries of facilitation is noteworthy since legumes are known for both their key role in many facilitative plant**−**plant interactions (Loreau and Hector 2001; Wright et al. 2017), specifically through the facilitative effects of older, established plants on neighbouring plants via nitrogen fixation and the related increase in resource availability (Wright et al. 2014).

### Caveats and future directions 

Although our artificial vegetation treatment provided a way to partially disentangle effects of plant**−**plant interactions above and below-ground, challenges remain. Importantly, while the treatment effects suggest positive effects of aboveground vegetation, presumably reflecting microclimate amelioration, it does not remove competition for light, and therefore will underestimate positive aboveground effects if light is limiting. One way to further isolate these effects might be to provide supplementary light to remove any light limitation (Hautier et al. 2009). Similarly, the treatment removes belowground interactions, revealing the negative effect of belowground competition. However, it also removes possible positive effects of belowground plant biomass, for example on nutrient availability and soil moisture retention (Anthelme et al. 2012). These effects might be simulated by manipulating soil organic matter content or nutrient conditions. Alternative approaches might be to kill vegetation, retaining its structural properties but excluding competitive (and facilitative) effects of living plant tissue, at least belowground. We can also expect other possible artefacts of artificial vegetation, such as shifts in the ratio of red:far-red light reaching seeds/seedlings, which could also influence germination and establishment rates. Consequently, we are not able to definitively say whether the benefits of artificial vegetation stem from the retention of facilitative effects aboveground, or the loss of competitive effects belowground. Nonetheless, despite these limitations our approach clearly demonstrates the action of both positive and negative effects of plant**−**plant interactions in these communities, both at high and low elevations, and shows the potential of creative experimental interventions to further unravel these effects.

In addition to the previous suggestions, future work may focus on the density-dependent effects of neighbours on focal plant performance, leading to a more nuanced understanding of neighbour densities associated with a switch from positive to negative effects. So far, we are only aware of demonstrations of how focal plant density (i.e. the facilitated species) can impact the performance of facilitator species (Schöb et al. 2014; García et al. 2016; Michalet et al. 2016). We hypothesise that reciprocal nonlinear effects also exist, such that low densities of surrounding species facilitate plant establishment (e.g. through microsite amelioration), while high densities lead to net competitive effects. Local variation in the composition of the background community might also have generated variation in establishment success within the natural vegetation treatment, since different species (and even individuals within species) can differ in their properties as nurses (Anthelme et al. 2017). We do not expect, however, that any variation of this sort would affect our interpretation of differences between our experimental treatments. Somewhat related to this, in our experiment we did not thin-down seedlings of focal individuals at each station, so that seedlings experienced (variable) intraspecific effects which might also have had negative and/or positive impacts on establishment. These intraspecific effects would also be interesting to examine in greater detail. Finally, we investigated seedling establishment as a key vital rate mediating population dynamics. Recruitment might be especially sensitive to competition and facilitation, due to small plant size increasing sensitivity to environmental conditions and to asymmetric competition (Goldberg et al. 1999; DeMalach et al. 2019; Klanderud et al. 2021). Later life stages (e.g. the growth, reproduction and survival of adult plants) might reveal a different balance of competitive and facilitative effects (Lyu and Alexander 2023). Work supporting the stress gradient hypothesis has often focused on adult plants (Brooker et al. 2008), potentially going some way to explaining the discrepancy with our results.

Lastly, our experiment was only conducted in two sites at contrasting elevations, so conclusions related to the effect of elevation on the net effect of plant**−**plant interactions must be interpreted with caution. Furthermore, although our high elevation site is located in treeless vegetation above timberline, it is below climatic treeline and so in the subalpine zone (Körner 2021). Previous work supporting the stress gradient hypothesis has typically been conducted at higher elevation alpine sites, where vegetation cover can be more sparse, environmental conditions more extreme and facilitative effects more pronounced (e.g.; Callaway et al. 2002).

### Implications for species range dynamics

Our results demonstrate the combined role of competition and facilitation by natural vegetation on high and low elevation plant establishment, with several implications for how these communities might change in a warming world. For example, climate change is causing species to on average shift their ranges upwards along elevation gradients. Our results are consistent with other work highlighting significant complexity in these range shifts, including large variation in the direction and magnitude of shifts and possible lags in the pace of shifts relative to the pace of climate change (Alexander et al. 2018; Rumpf et al. 2018). For example, several low elevation species were able to establish at the high elevation site today, even at rates exceeding those of resident high elevation species, yet are not yet present within the high elevation community. One possible explanation could be that low elevation species are dispersal limited and have not yet naturally reached the high elevation site. However, we hypothesise that these species (currently found only several 100’s of metres away) may experience strong competition from the resident vegetation that limits their establishment when they do periodically disperse there. This is in keeping with other recent work involving some of the same study species, which has demonstrated an important role for competition at high elevation range limits (Lyu and Alexander 2022). Our results also suggest that disturbances, which may create gaps within closed high elevation vegetation and allow establishment (Lembrechts et al. 2016), may especially benefit large-seeded species, whose seed provisioning putatively buffers against harsh microclimates in vegetation gaps. Finally, our results suggest that facilitation, commonly thought to influence plants most strongly at their upper elevation range limits, might often play a stronger role at lower elevations than commonly assumed. It is of course possible that facilitation is still pivotal for the establishment of small-seeded species or for species establishing at even higher elevations than our field site (Choler et al. 2001; Batllori et al. 2009; Barbeito et al. 2012; Ameztegui and Coll 2013; Stephan et al. 2021). In sum, further disentangling the positive and negative components of plant**−**plant interactions should help us to develop a more nuanced understanding of, and potentially a better ability to predict, how species interactions mediate climate change impacts on plant communities.

## Supplementary Information

Below is the link to the electronic supplementary material.

Supplementary file1 (TXT 305 KB)

Supplementary file2 (CSV 5467 KB)

Supplementary file3 (CSV 165 KB)

Supplementary file4 (CSV 126 KB)

Supplementary file5 (R 58 KB)

Supplementary file5 (DOCX 3744 KB)

## Data Availability

Raw data and code associated with this article are provided in Supplementary Information.
