# Supplementary material for: Positive and negative plant−plant interactions influence seedling establishment at both high and low elevations
Source: Alp Bot. 2023 Nov 24;134(1):15–27. doi: 10.1007/s00035-023-00302-8 (PMC11219458; doi:10.1007/s00035-023-00302-8)
Supplement: Supplementary file 6 — Supplementary file5 (DOCX 3744 KB) [file 35_2023_302_MOESM6_ESM.docx]

# SUPPORTING INFORMATION

**Table S1.** Selected plant species, indicating their plant families, functional groups, elevation origin and average seed mass.

| **Plant Species** | **Plant Family** | **Functional group** | **Origin** | **Seed mass (mg)** |
| --- | --- | --- | --- | --- |
| *Plantago media* | Plantaginaceae | forb | low | 0.356 |
| *Plantago atrata* | Plantaginaceae | forb | high | 2.590 |
| *Scabiosa columbaria* | Caprifoliaceae | forb | low | 1.612 |
| *Scabiosa lucida* | Caprifoliaceae | forb | high | 1.536 |
| *Bromus erectus* | Poaceae | grass | low | 4.418 |
| *Poa alpina* | Poaceae | grass | high | 0.432 |
| *Brachypodium pinnatum* | Poaceae | grass | low | 2.506 |
| *Sesleria caerulea* | Poaceae | grass | high | 1.543 |
| *Medicago lupulina* | Fabaceae | legume | low | 1.391 |
| *Lotus alpinus* | Fabaceae | legume | high | 1.172 |

| **Table S2.** Environmental parameters for every treatment and site. Data are mean values per treatment and site ± standard error. | | | | | | |
| --- | --- | --- | --- | --- | --- | --- |
| **Environmental Parameter** | **Low elevation** | | | **High elevation** | | |
|  | bare soil | artificial vegetation | natural vegetation | bare soil | artificial vegetation | natural vegetation |
| Mean daily soil temperature over the vegetation period [°C] | 15.61 ± 0.07 | 13.98 ± 0.04 | 13.98 ± 0.04 | 11.48 ± 0.06 | 10.37 ± 0.04 | 10.86 ± 0.03 |
| Mean soil moisture measured on one day (02.09.2021) [% VWC] | 19.31 ± 0.36 | 22.43 ± 0.51 | 34.51 ± 0.52 | 26.70 ± 0.37 | 29.92 ± 0.40 | 35.78 ± 0.53 |
| Mean light reaching the ground [µmol m^-2^ s^-1^] | 1425.33 ± 10.56 | 56.79 ± 3.66 | 26.54 ± 2.12 | 1406.28 ± 23.70 | 61.21 ± 3.44 | 36.29 ± 3.05 |

**Table S3.** Results of linear mixed effects models describing differences in environmental parameters (soil temperature over the course of the experiment; soil volumetric water content on 02.09.2021; light interception by the vegetation on 01.09.2021) between experimental sites (low *vs* high elevation), vegetation treatment (natural vegetation, artificial vegetation and bare soil) and their interaction. The bare soil treatment was excluded in the light-interception model. The temperature model included the identity of the temperature logger (two loggers per treatment and site) as a random effect; the soil moisture and light interception models included experimental plot as a random effect, with the latter model also including experimental block as a random effect. Soil temperature and light interception were square-root transformed to satisfy model assumptions.

| **Response variable** | **Fixed effect** | **χ^2^** | **df** | **P** |
| --- | --- | --- | --- | --- |
| Soil temperature (°C) | Treatment (T) | 18.03 | 2 | <0.001 |
|  | Site (S) | 379.94 | 1 | <0.001 |
|  | T x S | 4.43 | 2 | 0.109 |
| Soil volumetric water content (%) | Treatment (T) | 146.11 | 2 | <0.001 |
|  | Site (S) | 40.01 | 1 | <0.001 |
|  | T x S | 11.71 | 2 | 0.003 |
| Light interception (%) | Treatment (T) | 57.37 | 1 | <0.001 |
|  | Site (S) | 1.47 | 1 | 0.226 |
|  | T x S | 0.41 | 1 | 0.520 |

**Fig. S1**. Environmental parameters during the experiment. Mean daily (a) and grand mean (b) soil temperature from 18 June to 25 October, and mean soil moisture measured as volumetric water content at a single day (02.09.2021) (c) for all treatments at the low (1400 m a.s.l.) and high (2000 m a.s.l.) elevation sites.

| 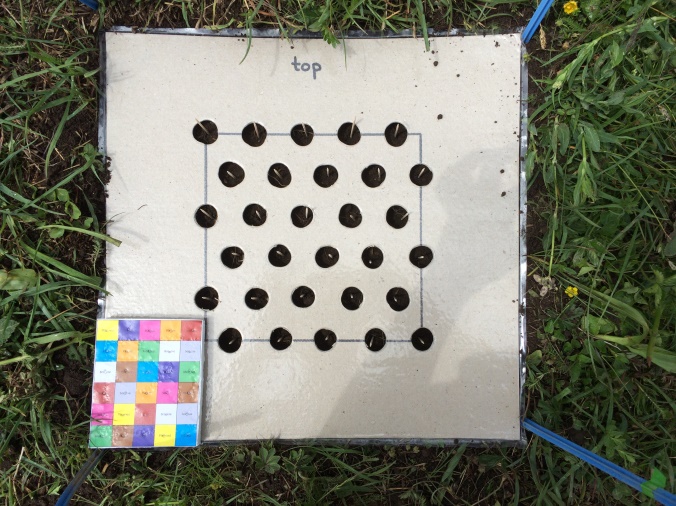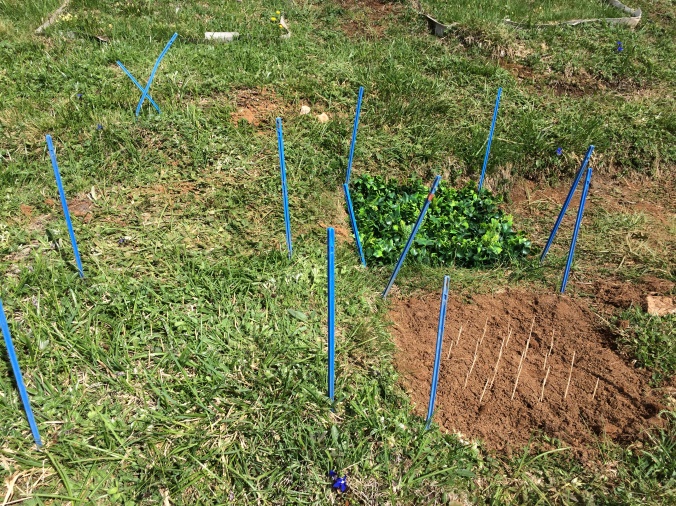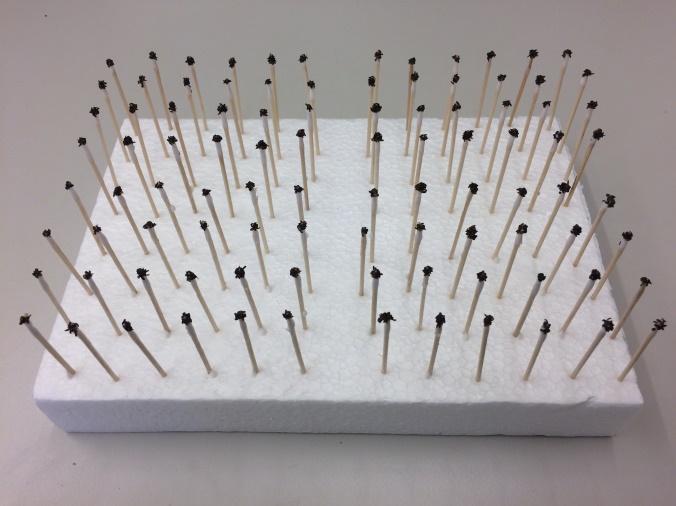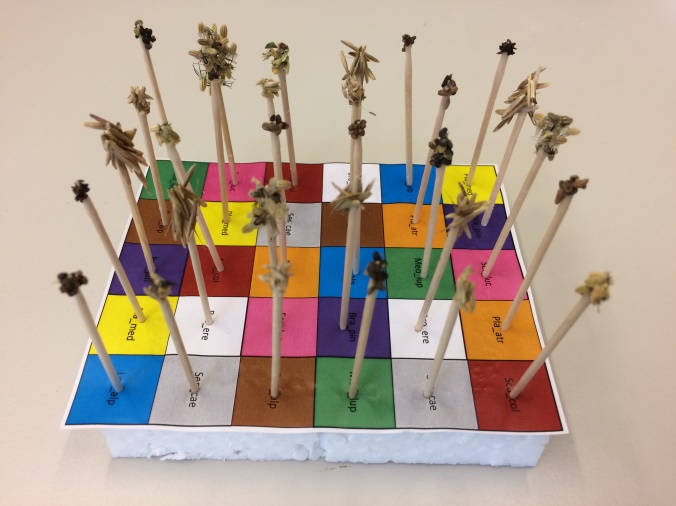 **(a)**  **(b)**  **(c)**  **(d)** |
| --- |

**Fig. S2.** Method for planting seeds of our focal species at specified locations within experimental plots. Toothpicks were dipped into tubes containing diluted water-soluble PVA glue and subsequently into tubes containing seeds of one focal plant species, resulting in an approximately equal amount of seeds of that species glued onto each toothpick (a). This procedure was repeated for each focal species. Positions of the plant species (i.e. the toothpicks with the seeds of the corresponding species) within a plot were randomised and the toothpicks inserted into a polystyrene template (b) before being planted into the field (c) into the plots of each treatment within a block (d; green plot bottom left: natural vegetation treatment, light brown plot bottom right: bare soil treatment, bright green plot top right: artificial vegetation treatment).

| 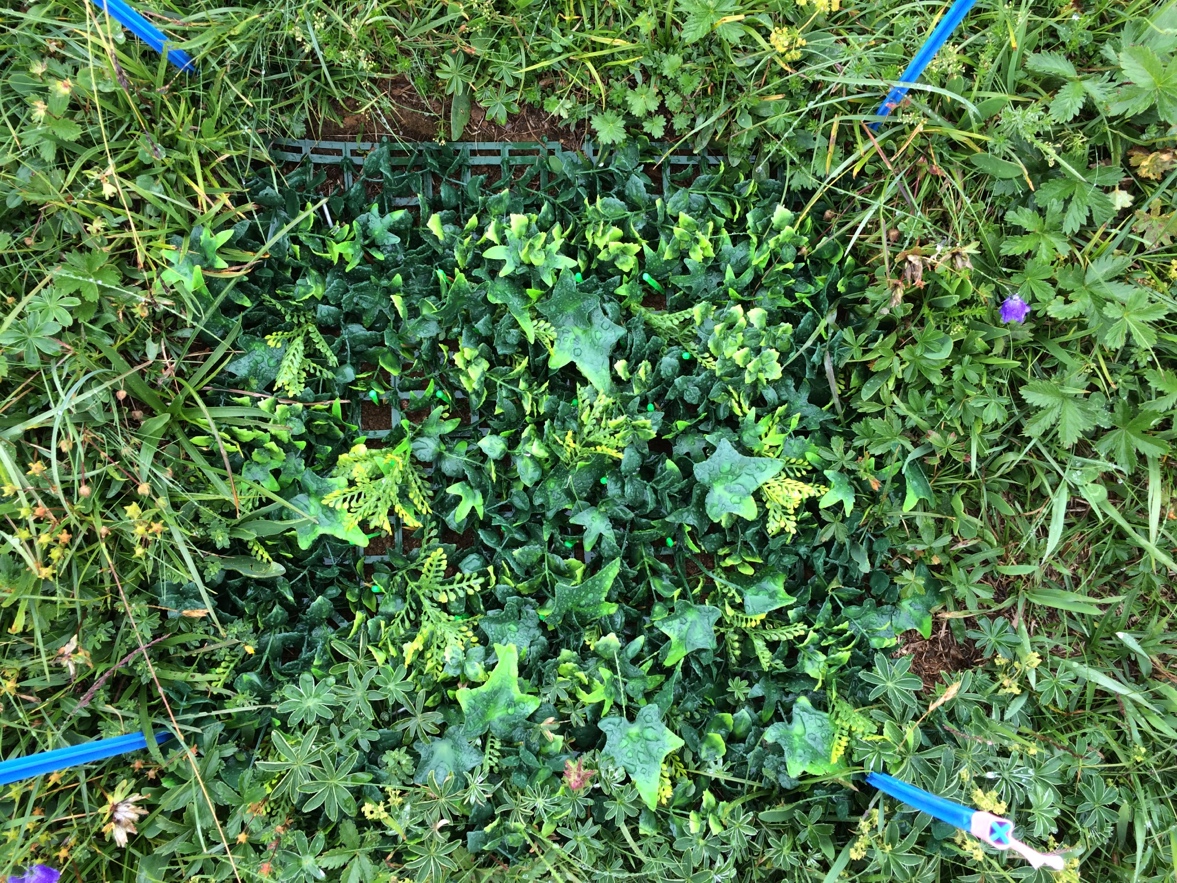 |
| --- |
| 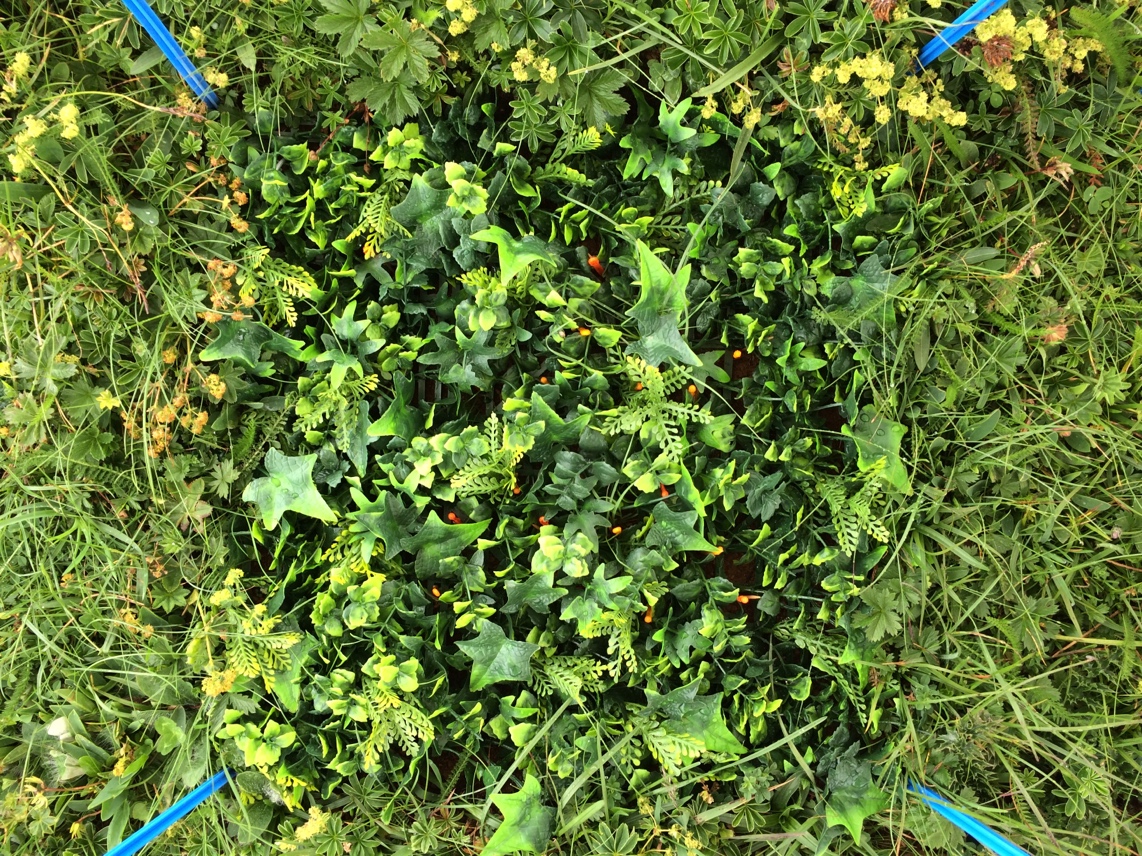 |

**Fig. S3.** Close-up photos of two artificial vegetation mats. Note the plastic grid supporting the artificial plants, into which the seeds of focal plants were planted, visible at the bottom of the top image. Note also the orange markers visible in the lower image, which were placed to aid locating the emerging seedlings.
